# Surgical management in immunosuppressed patients with sigmoid diverticulitis, still a challenge: a single-center observational study

**DOI:** 10.1007/s00384-022-04226-3

**Published:** 2022-08-02

**Authors:** Sascha Vaghiri, Dimitrios Prassas, Wolfram Trudo Knoefel, Andreas Krieg

**Affiliations:** Department of Surgery (A), Heinrich-Heine-University and University Hospital Duesseldorf, Duesseldorf, Germany

**Keywords:** Immunosuppression, Sigmoid diverticulitis, Emergency and elective sigmoidectomy, Morbidity and mortality rates

## Abstract

**Purpose:**

The question of whether immunosuppressed (IS) patients should be offered elective sigmoidectomy following a single episode of diverticulitis is controversial. We intended to examine the perioperative outcome of IS and immunocompetent (IC) patients after sigmoid resection.

**Methods:**

A single institutional cohort study was conducted, including all surgically treated patients with sigmoid diverticulitis between 2004 and 2021. IS and IC patients were further subdivided into emergency and elective cases. Morbidity and mortality in both groups and factors influencing surgical outcome were examined using uni- and multivariate regression analyses.

**Results:**

A total of 281 patients were included in the final analysis. Emergency surgery was performed on 98 patients while 183 patients underwent elective sigmoid resection. Emergency sigmoidectomy demonstrates significantly higher morbidity and mortality rates in IS patients as compared to IC patients (81.81% vs. 42.1%; *p* = 0.001, respectively 27.27% vs. 3.94%; *p* = 0.004), while major morbidity and mortality was similar in both groups in the elective setting (IS: 23.52% vs. IC: 13.85%; *p* = 0.488, respectively IS: 5.88% vs. IC: 0%; *p* = 1). On multivariate regression analysis for major postoperative morbidity, ASA score [OR 1.837; (95% CI 1.166–2.894); *p* = 0.009] and emergency surgery under immunosuppression [OR 3.065; (95% CI 1.128–8.326); *p* = 0.028] were significant. In-hospital mortality was significantly related to age [OR 1.139; (95% CI 1.012–1.282); *p* = 0.031], preoperative CRP count [OR 1.137; (95% CI 1.028–1.259); *p* = 0.013], and immunosuppression [OR 35.246; (95% CI 1.923–646.176), *p* = 0.016] on multivariate analysis.

**Conclusions:**

Elective surgery for sigmoid diverticulitis in immunocompromised patients demonstrates higher efficacy and safety when compared to sigmoid resection in the emergency setting.

## Introduction

Sigmoid diverticulitis is a very common condition in western countries, increasingly affecting younger patients besides the elderly population [1, 2]. With 300,000 hospital admissions per year in the US, the annual health care costs are exceedingly high as reported by large epidemiological studies bringing along a considerable socio-economic burden [3–5]. The diversity of sigmoid diverticulitis is reflected by the broad range of clinical manifestations, from mild inapparent disease stages to complicated forms yielding in free perforation [6, 7].

About 1–3.5% of transplanted and immunosuppressed patients develop acute sigmoid diverticular disease [8, 9]. As the total number of patients under immunosuppression for various reasons is expected to rise continuously diverticular disease will become increasingly important. Sigmoid diverticulitis is especially challenging in this subgroup because impaired immune response capacity results in delayed diagnosis and thus adequate treatment predisposes to an unfavorable outcome [10–12].

IS patients suffering from sigmoid diverticulitis are reportedly more prone to experience an initial severe disease course with abscess formation and perforation [13, 14]. The overall mortality in IS patients is 25% and about 56% if treated only nonoperatively, as opposed to 2.6–6% in the general population with acute diverticulitis requiring hospitalization [8, 15, 16]. Accordingly, a low threshold for elective sigmoid resection has been advocated for a long time to prevent potential further complicated disease recurrence after cessation of an acute episode in IS patients [11, 17, 18]. This approach is recently challenged by data demonstrating comparable recurrence rates in IS and IC patients after successful medical therapy without the necessity of subsequent emergent surgery in the IS cohort, outbalancing significant major morbidity in IS patients when undergoing elective sigmoidectomy [19–21]. Furthermore, conservative management of uncomplicated diverticulitis in transplanted patients was not associated with inferior clinical outcomes in comparison to immunocompetent patients [22]. In light of the above, the latest American Society of Colon and Rectal Surgeons (ASCRS) guidelines propose an individualized treatment strategy and only surgery in those IS patients with inevitable resection indications [23].

In an attempt to shed more light on the above mentioned discrepancy in the literature, the present study was conducted to compare and evaluate the postoperative outcome in IS and IC patients in the elective and emergency setting, respectively. Potential factors of an eventful outcome and mortality were elucidated across the whole study population.

## Material and methods

This study was conducted after approval of the institutional ethical board of the Medical Faculty, Heinrich-Heine University, Dusseldorf, Germany (study-no.: 2021–1346), in accordance with the latest version of the Declaration of Helsinki and its amendments.

All patients who were treated with acute or chronic diverticulitis at our department from January 2004 to July 2021 were enrolled and retrospectively analyzed. Patients were excluded if they solely received nonsurgical management. Relevant information was retrieved from our prospectively maintained clinical database. These include demographics [age, gender, and body mass index (BMI)], number of previous attacks, clinic and symptoms at admission, radiological evaluation, laboratory parameters (inflammatory and organ specific parameters), relevant comorbidities and medication, American Society of Anesthesiologists (ASA) score, current immune status, reason and type of immunosuppression, preoperative medical course, detailed medical and surgical treatment strategy, surgical procedures, conversion rate, ostomy creation, ostomy reversal rate, perioperative minor and major complications and mortality (wound infection, burst-abdomen, gastrointestinal or urological leakage, anastomotic stenosis, mechanical or paralytic ileus, intra-abdominal abscess formation, pneumonia, and cardiovascular complications), redo surgery or intervention, graft function, postoperative length of hospital stay (LOS), and outpatient follow-up.

Diagnosis of sigmoid diverticulitis was made based on clinical and radiological assessment at the first hospital presentation. We routinely applied abdominopelvic CT-imaging in cases of suspicious diverticular disease or in the diagnostic work-up of severe abdominal complaints with elevated inflammatory markers. Diverticular disease stage evaluation was based on radiological imaging and intraoperative finding according to the Classification of Diverticular Disease (CDD) [24]. Postoperative complications were rated using the Clavien-Dindo Classification [25] and major morbidity was defined as Clavien-Dindo ≥ 3b.

All medical or surgical treatment strategies were consistent with the current established German guidelines for diverticular disease [24]. Failed initial medical treatment and clinical deterioration, as well as free perforation, prompted urgent or emergent sigmoidectomy during the first hospitalization. Elective surgery was performed either within 6 weeks after the acute attack (early elective) or after 6 weeks in the inflammation-free interval (delayed elective) The surgical approach (open vs. laparoscopic) was primarily determined based on the severity of the diverticular disease stage, the clinical condition of the patient and contraindications of laparoscopic surgery and secondly at the discretion of the involved surgeon. The technique of laparoscopic sigmoid resection was described in our previous study [26]. Patients with fecal or purulent contamination and severe sepsis underwent damage control sigmoidectomy and subsequent second or third look procedures if necessary. The study cohort was further divided into two groups based on the immunological status at the time point of hospital admission: immunocompetent (IC) or immunosuppressed (IS). The IS population included patients with solid organ transplantation, acquired or congenital immune deficit syndromes, and autoimmune diseases requiring immunosuppressive medication. Perioperative discontinuation, dose change, or maintenance therapy in each patient was determined interdisciplinary.

The primary outcome of interest was major morbidity and mortality in IC and IS patients and the further evaluation of their prevalence within the elective and emergency setting. Furthermore, factors influencing in-hospital morbidity and mortality were evaluated.

### Statistical analysis

Statistical analysis was performed using SPSS 23.0 software (IBM Corp., Armonk, NY). Continuous variables, expressed as median and interquartile range (IQR), were compared using either the Mann–Whitney *U* test or the *t*-test. Therefore, we first tested the continuous variables for normal distribution. For normally distributed data, the *t*-test was used, while the Mann–Whitney *U* test was preferred for data that did not have a normal distribution. Categorical variables were summarized as frequencies (%) and compared using Fisher’s exact or chi-square test. Risk factors for in-hospital morbidity and mortality were identified using univariate analysis. Variables with *p* < 0.1 were included in the multivariable logistic regression. The backward stepwise selection was used to create a final model. In all analysis, a *p*-value of < 0.05 indicated statistical significance.

## Results

### Patient characteristics

During the study period, 281 patients with acute or chronic diverticulitis underwent sigmoidectomy at our department. Thirty-nine (13.8%) IS patients and 242 (86.2%) IC patients meet the inclusion criteria. The IS cohort consisted of 15 patients with renal transplantation (38.5%), one patient with combined renal and cardiac transplantation (2.6%), three patients having human immunodeficiency virus (HIV) (7.7%), one patient with metastatic breast cancer undergoing chemotherapy (2.6%), and 19 patients with the autoimmune disease taking oral corticosteroids (48.7%). All patients with autoimmune disease who were taking corticosteroids in any subform for at least 4 weeks before their surgical treatment in our department were included in our analysis. The lowest cortisone dose in our patient collective was 5 mg prednisolone equivalent/day. Besides corticosteroids, concomitant non-corticosteroid medication was recorded in 8 patients (e.g., methotrexate, cyclosporine, azathioprine, cyclophosphamide, and rituximab). In transplant patients, immunosuppression was maintained perioperatively with tacrolimus in addition to corticosteroids and mycophenolic acid if indicated.

Patients’ demographics were similar except for a lower BMI in the IS group (*p* > 0.001) (Table 1). IS patients had significantly higher ASA scores (*p* > 0.001) and a higher rate of chronic renal insufficiency (*p* > 0.001). The CDD distribution including complicated cases (CDD 2a, 2b, and 3c) was equal in both groups (*p* = 0.177). IS patients were admitted after a median of 1.75 (1-2) previous attacks and IC patients 1.88 (1-2.75), respectively (*p* = 0.664). Preoperative inflammatory markers did not differ between both groups (*p* > 0.05). Emergency surgery was significantly more necessary in IS patients in comparison to IC patients (56.41% vs. 30.89%, respectively, *p* = 0.003). Of the 22 IS patients undergoing emergent sigmoidectomy, failed initial medical treatment was observed in five cases (22.72%).

**Table 1:** Patient characteristics

	**IS (*n* = 39)**	**IC (*n* = 242)**	***P***
**Age, median (IQR)**	60 (49–72)	59 (49–69)	0.595
**Gender (M/F)**	M: 22/F: 17	M:118/F: 124	0.394
**BMI, median (IQR)**	24.73 (22.24–26.78)	27.86 (24.07–31.21)	** > 0.001**
**ASA, median (IQR)**	2.85 (3–3)	2.17 (2–3)	** > 0.001**
**DM Type II (*n*; %)**	4 (10.25)	18 (40)	0.517
**Hypertension (*n*; %)**	26 (66.66)	111 (45.86)	0.014
**Chronic renal insufficiency (*n*; %)**	19 (48.72)	11 (4.47)	** > 0.001**
**CDD classification (*n*; %)**			0.177
Ia	0	1 (0.4)	
Ib	6 (15.4)	44 (18.2)	
IIa	9 (23.1)	79 (32.6)	
IIb	4 (10.3)	28 (11.6)	
IIc	15 (38.5)	65 (26.9)	
IIIa	0	1 (0.4)	
IIIb	2 (5.1)	1 (0.4)	
IIIc	3 (7.7)	23 (9.3)	
**CRP (mg/dl), median (IQR)**	5.9 (1.1–20.7)	4.7 (0.5–14.1)	0.218
**WBC (× 1000/µl), median (IQR)**	10.65 (6.1–12.3)	11.36 (6.9–15.6)	0.490
**Emergency urgery (*n*; %)**	22 (56.41)	76 (30.89)	**0.003**
**Previous diverticulitis episodes, median (IQR)**	1.75 (1–2)	1.88 (1–2.75)	0.664

*IQR* interquartile range, *BMI* body mass index, *ASA score* American Society of Anesthesiologists, *CRP* C-reactive protein, *WBC* white blood cells

### Operative data and outcome

A primarily laparoscopic approach was significantly more frequent in the IC group (53.3% vs. 15.38%, *p* > 0.001) (Table 2). In both groups the conversion rates were similar if the procedure was performed laparoscopically (IS 7.69% vs. IC 10.33%, *p* = 0.778). Sigma resection in IS patients was associated with a nearly two fold increase of ostomy creation in comparison to the IC group (protective ostomy IS: 20.51% vs. IC 8.26%; *p* = 0.018, end colostomy IS 46.15% vs. IC 26.03%, *p* = 0.013).

**Table 2:** Operative data and outcome

	**IS (*n* = 39)**	**IC (*n* = 242)**	***P***
**Primarily laparoscopic (*n*; %)**	6 (15.38)	129 (53.3)	** > 0.001**
**Conversion to open (*n*; %)**	3 (7.69)	25 (10.33)	0.778
**Hartmann resection (*n*; %)**	18 (46.15)	63 (26.03)	**0.013**
**Protective ostomy (*n*; %)**	8 (20.51)	20 (8.26)	**0.018**
**Primary anastomosis without ostomy (*n*; %)**	13 (33.33)	159 (65.7)	** > 0.001**
**Overall morbidity (*n*; %)**	29 (74.36)	97 (40.08)	** > 0.001**
**Wound infection (*n*; %)**	21 (53.84)	68 (28.09)	** > 0.001**
**Fascia insufficiency (*n*; %)**	4 (10.26)	9 (3.71)	0.090
**Anastomotic leak (*n*; %)**	3/21 (14.29)	8/179 (4.47)	0.095
**Postoperative ileus (*n*; %)**	2 (5.13)	7 (2.89)	0.703
**Intraabdominal abscess (*n*; %)**	0	2 (0.82)	0.740
**Clavien-Dindo ≥ 3b (*n*; %)**	16 (41.03)	41 (16.94)	** > 0.001**
**Revisional surgery (*n*; %)**	11 (28.2)	39 (16.11)	0.075
**Mortality (*n*; %)**	7 (17.95)	3 (1.23)	** > 0.001**
**Stoma reversal (*n*; %)**	15/26 (57.69)	60/83 (72.29)	0.160
**Operative time (min), median (IQR)**	211.35 (173.75–238.50)	262.79 (205.0–312.50)	** > 0.001**
**Length of hospital stay (days), median (IQR)**	26.84 (17.0–45.0)	14.45 (8.0–18.25)	**0.016**

Overall morbidity was significantly higher in IS patients as opposed to IC patients (74.36% vs. 40.08%, *p* > 0.001). Moreover, postoperative wound infection was found to be significantly higher in the IS group in comparison to IC patients (53.84% vs. 28.09%; *p* > 0.001). The rates of fascia insufficiency (*p* = 0.090), anastomotic leak (*p* = 0.095), postoperative ileus (*p* = 0.703) and intra-abdominal abscess formation (*p* = 0.740) were not different comparing IS and IC patients as whole. Nevertheless, a significantly higher rate of major postoperative complications (Clavien-Dindo ≥ 3b) was observed in the IS group (41.03% vs. 16.94%; *p* > 0.001), while the necessity of revisional surgery was equally contributed (IS: 28.2% vs. IC: 16.11%; *p* = 0.075). IS patients had a significantly shorter operative time and a longer hospital stay compared to the IC group [IS: 211.35 (173.75–238.50) min vs. IC: 262.79 (205.0–312.50) min; *p* > 0.001, respectively, IS: 26.84 (17.0–45.0) days vs. IC: 14.45 (8.0–18.25) days; *p* = 0.016]. Finally in-hospital mortality was significantly different between both groups [IS: 7 patients (17.95%) vs. IC: 3 patients (1.23%); *p* > 0.001].

### Elective vs. emergency sigmoidectomy

We further subdivided the IS and IC groups with respect to the indication of surgery (elective vs. emergency). In the elective sigmoidectomy cohort, 17 IS, and 166 IC were included, while emergent sigmoid resection was performed in 22 IS and 76 IC patients (Tables 3 and 4). The rate of primarily laparoscopic sigmoid resection in the elective setting was significantly higher in favor of IC patients (IS: 35.29% vs. IC: 74.7%; *p* > 0.001). These patients had a higher rate of primary anastomosis without protective deviation as opposed to their IS counterparts (IC: 91.56% vs. IS 70.58%; *p* = 0.020). No significant differences in major complications (Clavien-Dindo ≥ 3b) (*p* = 0.488), wound infection (*p* = 0.220), fascia insufficiency (*p* = 1), anastomotic leak (*p* = 0.507), postoperative ileus (*p* = 1), intra-abdominal abscess formation (*p* = 1), revisional surgery (*p* = 1), hospital stay (*p* = 0.588), and overall mortality (*p* = 1) were observed. Overall morbidity was significantly higher in IS patients (IC 39.15% vs. IS 64.7%; *p* = 0.041). However elective sigmoid resection in IS patients was significantly shorter in duration as opposed to IC patients [IS: 221.13 (175.0–250.0) min vs. IC: 283.51 (229.25–325.0) min; *p* = 0.004]. In the scenario of an emergency sigmoid resection immunosuppression was associated with significantly increased rates of postoperative overall and major morbidity (Clavien-Dindo ≥ 3b) [IS: *n* = 18 (81.81%) vs. IC: *n* = 32 (42.1%); *p* = 0.001], respectively [IS: *n* = 12 (54.54%) vs. IC: *n* = 18 (23.68%); *p* = 0.006] and mortality [IS: *n* = 6 (27.27%) vs. IC *n* = 3 (3.94%); *p* = 0.004].

**Table 3:** Outcome of elective sigmoidectomy in immunocompromised and immunocompetent patients

	**Elective sigmoidectomy**		
	**IS (*n* = 17)**	**IC (*n* = 166)**	***P***
**Primarily laparoscopic (*n*; %)**	6 (35.29)	124 (74.69)	** > 0.001**
**Conversion (*n*; %)**	3/6 (50)	23/124 (18.55)	0.06
**Hartmann (*n*; %)**	0	3 (1.8)	1
**Protective ostomy (*n*; %)**	5 (29.41)	11 (6.62)	**0.009**
**Primary anastomosis without protective ostomy (*n*; %)**	12 (70.58)	152 (91.56)	**0.020**
**Overall morbidity (*n*; %)**	11 (64.7)	65 (39.15)	**0.041**
**Wound infection (*n*; %)**	7 (41.17)	45 (27,1)	0.220
**Fascia insufficiency (*n*; %)**	0	2 (1.2)	1
**Anastomotic leak (*n*; %)**	1/17 (5.88)	6/163 (3.68)	0.507
**Postoperative ileus (*n*; %)**	0	6 (3.61)	1
**Intraabdominal abscess (*n*; %)**	0	2 (1.2)	1
**Clavien-Dindo ≥ 3b (*n*; %)**	4 (23.52)	23 (13.85)	0.488
**Revisional surgery (*n*; %)**	2 (11.76)	23 (13.85)	1
**Mortality (*n*; %)**	1 (5.88)	0	1
**Stoma reversal (*n*; %)**	4/5 (80)	14/14 (100)	1
**Operative time (min), median (IQR)**	221.13 (175.0–250.0)	283.51(229.25–325.0)	**0.004**
**Length of hospital stay (days), median (IQR)**	14.7 (9.5–15.8)	13.20 (7.0–15.0)	0.588

**Table 4:** Outcome of emergency sigmoidectomy in immunocompromised and immunocompetent patients

	**Emergency Sigmoidectomy**		
	**IS (*n* = 22)**	**IC (*n* = 76)**	***P***
**Primarily laparoscopic (*n*; %)**	0	3 (3.94)	1
**Conversion (*n*; %)**	n/a	2/3 (66.66)	n/a
**Hartmann (*n*; %)**	18 (81.81)	60 (78.94)	1
**Protective ostomy (*n*; %)**	3 (13.63)	9 (11.84)	0.726
**Primary anastomosis without protective ostomy (*n*; %)**	1 (4.54)	7 (9.21)	0.679
**Overall morbidity (*n*; %)**	18 (81.81)	32 (42.1)	**0.001**
**Wound infection (*n*; %)**	14 (63.63)	23 (30.26)	**0.006**
**Fascia insufficiency (*n*; %)**	4 (18.18)	7 (9.21)	0.260
**Anastomotic leak (*n*; %)**	2/4 (50)	2/16 (12.5)	0.162
**Postoperative ileus (*n*; %)**	2 (9.09)	1 (1.31)	0.126
**Intraabdominal abscess (*n*; %)**	0	0	1
**Clavien-Dindo ≥ 3b (*n*; %)**	12 (54.54)	18 (23.68)	**0.006**
**Revisional surgery (*n*; %)**	9 (40.9)	16 (21.05)	0.060
**Mortality (*n*; %)**	6 (27.27)	3 (3.94)	**0.004**
**Stoma reversal (*n*; %)**	11/21 (52.38)	45/69 (65.21)	0.201
**Operative time (min), median (IQR)**	203.63 (160.0–225.0)	216.69 (160.0–245.0)	0.468
**Length of hospital stay (days), median (IQR)**	36.23 (14.0–47.75)	28.71 (12.0–36.75)	**0.027**

Univariate analysis of factors influencing major morbidity was significant for ASA score (*p* = 0.001), hypertension (*p* = 0.011), immunosuppression (*p* = 0.001), emergency surgery (*p* = 0.002), and emergency surgery under immunosuppression (*p* > 0.001). Among these factors, ASA score [OR 1.837; (95% CI 1.166–2.894); *p* = 0.009] and emergency surgery under immunosuppression [OR 3.065; (95% CI 1.128–8.326); *p* = 0.028] remained significant in the logistic regression analysis (Table 5). Univariate analysis for potential risk factors associated with in-hospital mortality revealed age (*p* = 0.01), ASA score (*p* = 0.024), hypertension (*p* = 0.017), preoperative CRP count (*p* = 0.003), renal failure (*p* < 0.001), immunosuppression (*p* < 0.001), emergency surgery (*p* < 0.001), and emergency surgery under immunosuppression (*p* < 0.001) as significant parameters. After multivariate regression analysis age [OR 1.139; (95% CI 1.012–1.282); *p* = 0.031], preoperative CRP count [OR 1.137; (95% CI 1.028–1.259); *p* = 0.013], and immunosuppression [OR 35.246; (95% CI 1.923–646.176); *p* = 0.016] are still considered important significant factors contributing to in-hospital mortality (Table 6).

**Table 5:** Uni- and multivariate analyses of factors affecting major morbidity

	**No major morbidity (n = 223)**	**Major morbidity (n = 57)**	**p**	**Odds ratio (95% CI)**	**p**
	*Univariate Analysis*			*Multivariate Analysis*	
**Gender (*n*; %)**			0.328		
* Male*	114 (51.1)	25 (43.9)			
* Female*	109 (48.9)	32 (56.1)			
**Age, median (IQR)**	58 (49–68)	62 (48–74)	0.065		
**BMI, median (IQR)**	27.60 (23.92–30.94)	26.85 (23.03–28.69)	0.377		
**ASA(*n*; %)**			**0.001**	1.837 (1.166–2.894)	**0.009**
* I*	30 (13.5)	3 (5.3)			
* II*	89 (39.9)	20 (35.1)			
* III*	55 (24.7)	16 (28.1)			
* IV*	4 (1.8)	7 (12.3)			
**CRP, median (IQR)**	8.53 (4.20–12.90)	11.06 (2.25–18.65)	0.116		
**WBC count, median (IQR)**	11.11 (6.90–15.35)	11.90 (5.97–15.10)	0.376		
**Operative time, median (IQR)**	256.10 (197.0–304.0)	256.67 (190.0–320.0)	0.959		
**Diabetes (*n*; %)**	17 (7.6)	5 (8.8)	0.782		
**Hypertension (*n*; %)**	100 (44.8)	36 (63.2)	**0.011**		
**Renal failure (*n*; %)**	20 (9)	10 (17.5)	0.058		
**Immunosuppression (*n*; %)**	23 (10.3)	16 (28.1)	**0.001**		
**Emergency surgery (*n*; %)**	68 (30.5)	30 (52.6)	**0.002**		
**Emergency surgery under immunosuppression (*n*; %)**	10 (4.5)	12 (21.1)	** > 0.001**	3.065 (1.128–8.326)	**0.028**

*BMI* body mass index, *ASA score* American Society of Anesthesiologists, *CRP* C-reactive protein, *WBC* white blood cells

**Table 6:** Uni- and multivariate analysis of factors affecting in-hospital mortality

	**No in-hospital mortality (*n* = 271)**	**In-hospital mortality (*n* = 10)**	***P***	**Odds ratio (95% CI)**	***P***
	*Univariate analysis*			*Multivariate analysis*	
**Gender (*n*;%)**			0.202		
* Male*	137 (50.6)	3 (30)			
* Female*	134 (49.4)	7 (70)			
**Age, median (IQR)**	59 (49–69)	70 (53–81.75)	**0.010**	1.139 (1.012–1.282)	**0.031**
**BMI, median (IQR)**	27.52 (23.79–30.82)	24.95 (22.42–27.37)	0.201		
**ASA (*n*; %)**			**0.024**		
* I*	33 (12.2)	0			
* II*	109 (40.2)	1/8 (12.5)			
* III*	65 (24)	6/8 (75)			
* IV*	10 (3.7)	1/8 (12.5)			
**CRP, median (IQR)**	8.71 (4.6–14.07)	21.21 (11.7–30.77)	**0.003**	1.137 (1.028–1.259)	**0.013**
**WBC count, median (IQR))**	11.26 (6.9–15.3)	11.20 (2.37–18.25)	0.977		
**Operative time, median (IQR)**	257.43 (195.0–305.0)	215.13 (167.5–242.5)	0.153		
**Diabetes (*n*; %)**	21 (7.7)	1/9 (11.1)	0.527		
**Hypertension (*n*; %)**	129 (47.6)	8/9 (88.9)	**0.017**		
**Renal failure (*n*; %)**	25 (9.2)	5/9 (55.6)	** < 0.001**		
**Immunosuppression (*n*; %)**	32 (11.8)	7 (70)	** < 0.001**	35.246 (1.923–646.176)	**0.016**
**Emergency surgery (*n*; %)**	89 (32.8)	9 (90)	** < 0.001**		
**Emergency surgery under immunosuppression (*n*; %)**	16 (5.9)	6 (60)	** < 0.001**		

*BMI* body mass index, *ASA score* American Society of Anesthesiologists, *CRP* C-reactive protein, *WBC* white blood cells

## Discussion

The results of our single institutional study demonstrate a significantly worse course in IS patients undergoing emergent sigmoidectomy in comparison to IC patients in terms of postoperative morbidity and mortality, while elective surgery in both groups displays similar outcome. Sigmoid diverticular disease has a high prevalence in western countries and, with an increasing number of even younger patients under immunosuppressive medication after transplantation or for autoimmune disease, this rate is expected to rise as immunosuppression bears a higher risk of acute diverticulitis [8, 9, 12]. For years, elective sigmoid resection has been advocated and incorporated in guidelines as the therapy of choice in sigmoid diverticulitis in order to avoid severe recurrent attacks necessitating emergency surgery [11, 17, 18, 27]. Sigmoidectomy in an emergency setting in IS patients is consistently associated with significantly higher morbidity and mortality rates throughout the literature as opposed to the normal population [1, 8, 12, 28, 29].

However, concerns regarding elective surgery arise from mainly four important factors: (1) sigmoid diverticulitis in IS patients is amenable to medical treatment [19, 21, 22, 30], (2) recurrence rates are identical between IS and IC patients [19, 21], (3) the course of recurrent sigmoid diverticulitis in both groups is similar with no significant disadvantages of IS patients [19, 30], and (4) elective sigmoidectomy under immunosuppression itself is associated with high morbidity rates [20, 21].

Samdani et al. [21] analyzed the outcome of 131 patients with and without chemotherapy (CTx) admitted for an acute episode of sigmoid diverticular disease. They found similar failure rates of medical treatment in both groups (CTx group 13.2% vs. non-CTx group 4.4%, respectively, *p* = 0.12), while disease severity at hospital presentation was equally distributed (*p* = 0.12). Moreover, the recurrence rate in both groups after medical management did not differ significantly (CTx group 20.5% vs. non-CTx group 18.5%). However, IS patients suffered more frequently a severe disease relapse compared to IC patients (87.5% vs. 29.4%, *p* = 0.01), requiring emergency surgery in 75% vs. only 23.5% in IC patients (*p* = 0.03). In another large retrospective study, 657 patients (107 immunosuppressed and 550 immunocompetent) with successful nonoperative management of their first acute episode were evaluated with a mean follow-up of 81.6 months [19]. Again, recurrence rates were similar in both groups (IS 21.5% vs. IC 20.5%, *p* = 0.82). Although IS patients with an advanced disease stage at initial presentation were more likely to have a complicated recurrent episode, the rate of emergent surgical intervention, however, was comparable. Interestingly, these results are in contrast to the findings of Klarenbeek and colleagues [27], who analyzed the course of 88 successful conservatively treated patients with acute diverticulitis. They found a fivefold higher risk of perforation at recurrence in a subgroup of 14 “high-risk” patients (36% vs. 7%, *p* = 0.002), which were defined as a combination of taking immunosuppressive medication, having chronic renal failure, or collagen vascular disease. A large nationwide database study with 26.987 included patients revealed a significantly higher rate of major morbidities in IS patients treated with an elective sigmoid resection compared to IC patients (OR, 1.46; 95% CI, 1.17–1.83) [20]. Recently, McKechnie et al. [31] performed a systematic review and meta-analysis addressing the outcome of IS patients in both emergency and elective surgery. In the 11 included studies, 2977 immunosuppressed patients and 780,630 immunocompetent patients were analyzed. Elective surgery under immunosuppression was associated with increased morbidity [RR 2.18, (95% CI 1.02–4.65), *p* = 0.04] while emergency surgery demonstrated a significant fatal outcome [RR 1.91, (95% CI 1.24–2.95), *p* < 0.01] in comparison to immunosufficient patients. These findings are in line with our results, as we found higher overall but not major morbidity rates in electively resected IS patients. However, in our study, emergency surgery concomitantly displayed a negative impact on both postoperative morbidity and mortality.

The comparable major morbidity rates after elective sigmoid resection between IC and IS patients in our analysis may be possibly attributed to the higher rate of protective ostomy creation in the IS subgroup (IS 29.41% vs. IC 6.62%, *p* = 0.009). IS patients demonstrate an anastomotic leakage rate of 6.8% in lower GI-tract surgery [32]. Therefore, primary anastomosis with deviation ostomy could be a reliable option in the setting of elective sigmoidectomy, preventing major morbidity [28].

The majority of our IS patients received perioperative corticosteroid monotherapy or combination therapy. Steroid intake has been implicated as a major risk factor for complicated diverticular diseases, e.g., perforation [11, 33]. Experimental studies demonstrated that increased oral glucocorticoid intake is significantly associated with elevated expression levels of matrix metalloproteinase-9 (MMP-9) through the activated glucocorticoid-induced tumor necrosis factor receptor (GITR) signaling pathway in activated CD68 + /CD163 + macrophages in tissue specimen of patients with complicated diverticulitis [34, 35]. Novel therapeutic strategies targeting the above-mentioned signaling pathway could be developed in severe diverticulitis stages with the dismal outcome under immunosuppressive medication to enhance therapy in the acute setting or even act as a prophylactic agent with regard to recurrent flares of the disease.

Finally, mortality following medical treatment in IS patients is reportedly as high as 56% [8], which practically means that more than half of these patients experience a fatal outcome during initial hospitalization or after disease recurrence if treated nonsurgically. Furthermore, emergency surgery under immunosuppression (*p* = 0.028) and immunosuppressive status per se (*p* = 0.028) were independently associated with major postoperative morbidity while, at the same time, IS patients, in general had a 35-fold increased probability of postoperative mortality (*p* = 0.016) in our multivariate analysis. These striking numbers highlight the need for an effective and individualized treatment strategy in this cohort. Therefore, higher overall morbidity rates after elective sigmoidectomy should be weighed against the risk of complicated disease relapse necessitating emergency sigmoid resection with a significantly inferior and potentially fatal outcome. This thesis opposes against the existing conjecture that elective sigmoidectomy in the IS patient subgroup should be avoided, as advocated by the latest American Society of Colon and Rectal Surgeons (ASCRS) guidelines [23].

Our study has some limitations with respect to its retrospective design and the possibility of Type II error occurrence. Notable difficulty and caution in interpreting the current data are accounted for by the relatively small sample size of the included IS patients and the heterogeneity of this subgroup encompassing patients with various indications for immunosuppression and differing immunosuppressive regimen. Furthermore, patient allocation in primary medical or surgical therapy and documentation of their respective course was not possible as only surgically treated patients were included. Potential selection bias could be considered by the fact that elective sigmoidectomy was offered more frequently to IS patients after the first diverticulitis episode [13].

## Conclusion

Elective sigmoidectomy in IS patients is justified considering significantly higher morbidity and mortality rates after emergency sigmoid resection at cost of increased overall but not major morbidity in the non-emergent setting. Primary anastomosis and deviation ostomy seems to be suitable approach. Further randomized trials are necessary to verify our findings.
